# White Blood Cell Count Measured Prior to Cancer Development Is Associated with Future Risk of Venous Thromboembolism – The Tromsø Study

**DOI:** 10.1371/journal.pone.0073447

**Published:** 2013-09-04

**Authors:** Kristine Blix, Hilde Jensvoll, Sigrid K. Brækkan, John-Bjarne Hansen

**Affiliations:** Hematological Research Group, Department of Clinical Medicine, University of Tromsø and Division of Internal Medicine, University Hospital of North Norway, Tromsø, Norway; Maastricht University Medical Center, Netherlands

## Abstract

**Background:**

Elevated white blood cell (WBC) count is associated with risk of venous thromboembolism (VTE) in cancer patients initiating chemotherapy. It is not known whether the risk of VTE by WBC count in cancer patients is causal or merely a consequence of the malignant disease. To address this question, we studied the association between WBC count, measured prior to cancer development, and risk of VTE in subjects who did and did not develop cancer during follow-up in a prospective population-based study.

**Methods:**

Baseline characteristics, including WBC and neutrophil counts, were measured in 24304 initially cancer-free subjects who participated in the Tromsø Study in 1994-1995. Incident cancer diagnosis and VTE events were registered up to September 1, 2007. In the cancer cohort, WBC and neutrophil counts were measured in average 7.1 years before cancer development. Cox-regression models were used to calculate hazard ratios (HRs) for VTE by WBC and neutrophil counts as categorized variables (<40^th^, 40-80^th^, and >80^th^ percentile) with 95% confidence intervals (CIs).

**Results:**

During follow-up, 1720 subjects developed cancer and there were 388 VTE events, of which 116 occurred in the cancer-group (6.9 per 1000 person-years) and 272 in the cancer-free group (1.1 per 1000 person-years). In those who developed cancer, WBC count above the 80^th^ percentile (≥8.6x10^9^ cells/L) was associated with a 2.4-fold higher risk (HR 2.36, 95% CI: 1.44-3.87) of VTE compared to WBC count below the 40^th^ percentile (<6.4x10^9^ cells/L). No association was found between WBC count and VTE in those who stayed cancer-free (HR 0.94, 95% CI 0.65-1.36). Similar findings were observed for neutrophils.

**Comment:**

Pre-cancer WBC count was associated with risk of VTE in cancer patients, but not in cancer-free subjects. Our findings suggest that leukocytes may play a causal role in cancer-related VTE rather than only reflecting the low-grade inflammation associated with cancer.

## Introduction

Venous thromboembolism (VTE), including deep vein thrombosis (DVT) and pulmonary embolism (PE), is a common disease with serious short- and long-term consequences. The overall incidence of VTE is 1-2 per 1000 person-years in adults, and it increases exponentially with higher age [1–3]. To date, many acquired and genetic risk factors for VTE have been identified, and cancer is established as one of the leading causes of VTE. Overall, cancer is associated with a 5-7 fold increased risk of VTE [4–6], and approximately 20% of the incident VTE events are associated with malignancy [1–3]. Cancer patients that develop VTE have shortened life-expectancy compared to cancer patients without VTE [7,8]. VTE in subjects with cancer also leads to frequent and prolonged hospitalizations, thereby consuming a substantial amount of health resources [9]. Furthermore, cancer patients with VTE more often suffer from recurrent VTE and bleeding complications during treatment with anticoagulants [10]. Current guidelines do not support thromboprophylaxis to all cancer patients, stressing the importance of identifying high-risk groups [11].

In population-based cohort studies, high leukocyte counts have been recognized as a risk factor for cancer mortality and arterial thrombosis [12–15]. Limited knowledge exists, however, on the association between white blood cell (WBC) count and risk of VTE. A population-based cohort study of cancer-free subjects reported no association between WBC count and risk of VTE [16]. In contrast, leukocytosis was identified as a risk factor for VTE and mortality in ambulatory cancer patients [17]. However, it is not known whether the risk of VTE by WBC count in cancer patients is causal, or merely a consequence of the malignant disease. The WBC count is often elevated in subjects with cancer [18], and leukocytes are shown to be important in cancer progression [19]. Accordingly, we found it pertinent to investigate whether WBC and neutrophil counts, the major subtype of WBC, measured prior to cancer diagnosis were associated with risk of VTE in cancer and cancer-free subjects recruited from a general population.

## Methods

### Ethics statement

The study was approved by the Regional Committee for Medical and Health Research Ethics in Northern Norway (REC North), and the participants gave their informed written consent to participate.

### Study population

Participants were recruited from the fourth survey of the Tromsø Study (conducted in 1994-95), a single-center, population-based cohort study. All inhabitants of the Tromsø municipality were invited and 77% (n=27 158) of the eligible population participated. Subjects who were no longer officially registered as inhabitants of Tromsø at baseline (n= 43), did not consent to medical research (n=201), had a known VTE prior to inclusion (n= 48), or missing values for total WBC or neutrophil count, (n=1679) were excluded from the study population. Furthermore, subjects with a history of cancer (n=753) or occult cancer at baseline (i.e. cancer diagnosis <1 year after baseline inclusion, n=130) were excluded to avoid influence of preexisting cancer on the WBC measurement. Subjects with non-melanoma skin cancers (ICD-7 codes: 191.0-191.9) were regarded as cancer-free subjects, due to the non-metastatic potential of the disease. Accordingly, the study population counted 24 304 subjects who were followed for a maximum of 12.9 years. Observational time was calculated for each subject from the date of enrollment to either the date of VTE, migration, death or end of follow-up (Sept 1, 2007), whichever came first. Subjects who got a diagnosis of cancer during follow-up and subjects who remained cancer-free were analyzed separately.

### Baseline measurements

Baseline data was obtained by clinical examination, non-fasting blood samples and self-administrated questionnaires. The blood samples were collected from an antecubital vein and analyses were performed by the Department of Clinical Chemistry, University Hospital, North Norway. For WBC and neutrophil counts, 5 ml of blood were collected into Vacutainer tubes containing EDTA as an anticoagulant (K3- EDTA 40 µL, 0.37 mol/L per tube), and analyzed within 12 hours by an automated blood cell counter (Coulter Counter®, Coulter Electronics, Luton, UK). Body weight and height were measured in subjects wearing light clothing and no shoes. Body mass index (BMI) was calculated as weight in kilograms, divided by the square of the height in meters (kg/m^2^). Information about physical activity level (average weekly hours of hard physical activity, i.e. activity causing sweating and/or breathlessness), current daily smoking (cigarettes/cigar/pipe), self-reported diabetes and history of cardiovascular disease [CVD] (angina pectoris, myocardial infarction or stroke) were obtained from the questionnaires.

### Cancer ascertainment

Surveillance of cancer diagnoses in the Norwegian population is performed by the Cancer Registry of Norway (CRN) and information about cancer in the cohort was obtained through linkage to the CRN. The CRN is considered a complete and valid registry, and in a recent evaluation of data quality it was found to have a completeness of 98.8% with 94% of the cases being histologically verified [20]. The CRN provided information about the date of diagnosis and location of the disease (ICD-7 codes 140-205).

### Venous thromboembolism ascertainment

First life time VTE events were identified by searching the hospital discharge diagnosis registry, the radiology registry and the autopsy registry at the University Hospital of North Norway, as previously described by Brækkan et al [1]. The University Hospital of North Norway is the only hospital that performs diagnostic verification and treatment of VTE in the region, and the discharge diagnosis registry includes both outpatient clinic visits and hospitalizations. A VTE event was recorded when all four of the following criteria were fulfilled; (i) objectively confirmed by diagnostic procedures (compression ultrasonography, venography, spiral computed tomography, perfusion-ventilation scanning, pulmonary angiography or autopsy); (ii) a diagnosis of deep vein thrombosis or pulmonary embolism made by the physician was written in the medical record; (iii) signs and symptoms consistent with VTE were present; and (iv) relevant treatment had been initiated (heparin, warfarin or similar agent, thrombolytics or vascular surgery). Registrations made from the autopsy registry required that VTE was the acknowledged cause of death or a significant contributor on the death certificate.

Provoking factors other than cancer were systematically obtained from the medical records of the VTE patients. Such factors were: recent surgery or trauma within the previous 8 weeks, acute medical conditions (acute MI, ischemic stroke or major infectious disease), marked immobilization (bed rest for > 3 days, wheelchair use, or long-distance travel exceeding 4 hours within the last 14 days prior to the VTE) or other provoking factors specifically described by a physician in the medical record (e.g. intravascular catheter).

### Statistical analyses

Linear regression and chi-square test were used in trend analyses of continuous and dichotomous baseline variables, respectively. Crude incidence rates of VTE and cancer were calculated across WBC categories in both cohorts. Cox proportional hazards regression models were used to determine hazard ratios (HR) with 95% confidence intervals. WBC and neutrophil counts were treated as continuous and categorized (<40^th^, 40-80^th^, and >80^th^ percentiles) variables. In the categorized analyses, the lower 40^th^ percentile was used as reference group. In the simple regression model HRs were adjusted for age and sex, whereas the multivariable model included age, sex, smoking, BMI, history of CVD, diabetes and hard physical activity. Multivariable adjusted associations between WBC count and risk of VTE in cancer and non-cancer subjects were visualized by generalized additive regression plots. In these plots, WBC counts (log transformed) were modeled with a 4-degrees of freedom smoothing spline fit in Cox proportional hazard models including the same variables as described above. The proportional hazards assumption was confirmed by evaluating the parallelism between the curves of the log-log survivor function. The statistical packages R (version 2.15.1 for windows) and SPSS (version 19, IBM SPSS Statistics) were used for the analyses.

## Results

In total, 1 720 subjects (7% of the population) were diagnosed with cancer during follow-up. Table 1 shows baseline characteristics for the cancer- and non-cancer cohort over categories of WBC count. In the cancer cohort (n=1 720), the mean age was higher (58 years) than in the non-cancer cohort (45 years). Age slightly decreased across increasing values of total WBC count in both cohorts, as did the proportion of subjects performing hard physical activity. Daily smoking was substantially more common across increasing categories of WBC count in both groups. Minor changes in BMI and no changes in history of CVD appeared across categories of WBC count.

**Table 1:** Baseline characteristics across categories of total white blood cell (WBC) count in subjects who developed cancer and subjects who did not develop cancer during follow-up.

	**Categories of WBC count (10^9^ cells/L)**			***P* for trend**
	**< 6.4**	**6.4-8.5**	**≥ 8.6**	
**Cancer**				
Subjects (n)	671	688	361	-
Age (years)	58.5 ± 13.3	58.2 ± 13.6	55.6 ± 12.8	0.002
Sex (female)	49.0 (329)	51.2 (352)	48.5 (175)	0.9
BMI (kg/m^2^)	25.9 ± 4.0	25.8 ± 4.2	25.3 ± 3.9	0.06
Daily smoking	21.0 (141)	44.0 (303)	70.6 (255)	<0.001
Physical activity (hard)	24.7 (164)	21.3 (145)	16.9 (60)	0.003
Self-reported DM	2.7 (18)	3.8 (26)	3.6 (13)	0.3
Self-reported CVD	11.2 (75)	12.4 (85)	9.4 (34)	0.5
**Non-cancer**				
Subjects (n)	8884	9214	4486	-
Age (years)	46.1 ± 14.9	45.7 ± 14.6	43.5 ± 13.1	<0.001
Sex (female)	49.7 (4414)	53.0 (4884)	54.7 (2455)	<0.001
BMI (kg/m^2^)	24.9 ± 3.6	25.3 ± 3.9	25.2 ± 4.0	<0.001
Daily smoking	19.0 (1689)	39.3 (3618)	66.0 (2963)	<0.001
Physical activity (hard)	35.3 (3121)	30.1 (2753)	27.4 (1218)	<0.001
Self-reported DM	1.3 (118)	1.9 (172)	1.4 (64)	0.3
Self-reported CVD	5.3 (475)	6.4 (592)	5.6 (252)	0.2

The Tromsø Study 1994-2007.

Values are given as mean ± 1 standard deviation or as percentages with absolute numbers in parentheses.

Physical activity (hard) ≥ one hour per week of activity that caused sweating or breathlessness.

BMI = Body mass index

DM = Diabetes mellitus

CVD = Cardiovascular disease

Throughout the study period 388 incident VTE events were registered, and 29% of the VTE events (n = 116) occurred in the cancer cohort. The clinical characteristics of VTEs occurring in the cancer- and non-cancer cohort are shown in table 2. The proportion of VTEs that could be related to one or more provoking factors other than cancer was similar in both cohorts (46% and 47%, respectively). Particularly, surgery was a predisposing event in the same proportion in cancer and non-cancer subjects. Traumas were more often associated with VTE in non-cancer patients, whereas other predisposing factors (i.e. intravascular catheters) more commonly occurred in cancer patients.

**Table 2:** Clinical characteristics of cancer and non-cancer related venous thromboembolism (VTE) events at the time of VTE diagnosis.

	**Cancer related VTE (n=116)**	**Non-cancer VTE(n=272)**
Total provoked^*^	45.7 (53)	47.4 (129)
Surgery^**^	15.5 (18)	18.4 (50)
Acute medical condition^†^	14.7 (17)	16.5 (45)
Trauma^**^	2.6 (3)	8.5 (23)
Immobilisation^‡^	19.0 (22)	17.6 (48)
Other provoking factor^§^	6.0 (7)	2.9 (8)

The Tromsø Study 1994-2007.

Values are given as percentages with absolute numbers in parentheses.

* One or more provoking factors, except for cancer.

** Within 8 weeks before the VTE event.

† Myocardial infarction, ischemic stroke or major infectious disease.

‡ Bed rest > 3 days, wheelchair, long haul travel >4 hours in the past 14 days.

§ Other provoking factor described by the physician, e.g. intravascular catheter.

**Figure 1:**
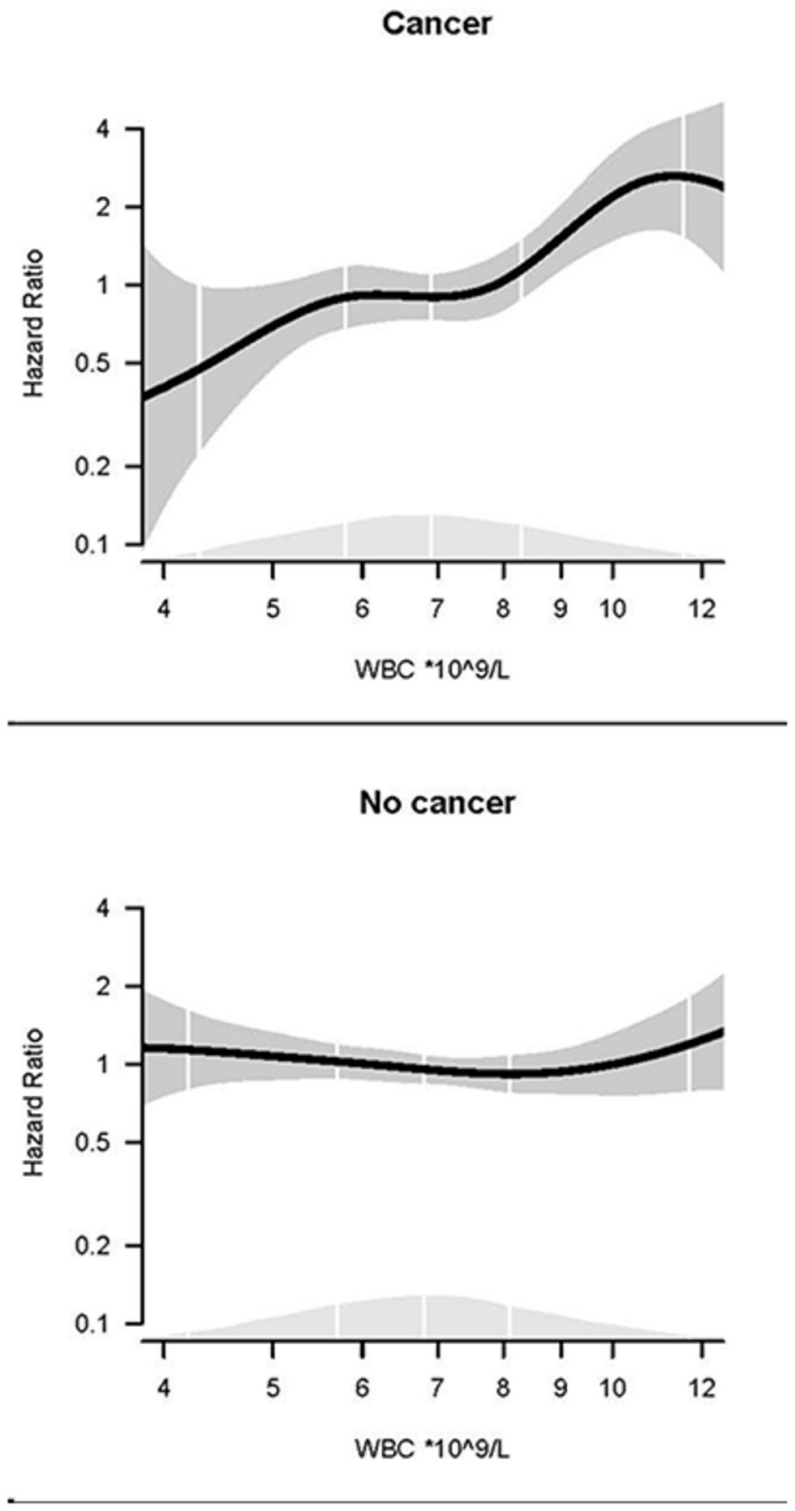
WBC count and risk of venous thromboembolism. Dose–response relationship between WBC count and risk of VTE in cancer and non-cancer subjects obtained by generalized linear regression. The regression models are adjusted for age, sex, BMI, smoking, self-reported diabetes, physical activity and self-reported CVD. The solid lines show HRs and the shaded areas show 95% CIs. Density plots show the distribution of WBC, and white vertical lines indicate 2.5^th^, 25^th^, 50^th^, 75^th^ and 97.5^th^ percentiles.

The risk of VTE by WBC and neutrophil counts in cancer and cancer free subjects are shown in table 3. The mean observational time was 9.8 years in the cancer cohort and 11.0 years in the non-cancer cohort. The overall crude incidence of VTE in the cancer cohort was 6.9 per 1000 person-years, compared to 1.1 per 1000 person-years in the non-cancer cohort (Table 3). In the cancer cohort, the VTE incidence increased from 6.2 per 1000 person-years in subjects with WBC count <6.4 x 10^9^ cells/L to 10.6 per 1000 person-years in subjects with total WBC count ≥8.6 x 10^9^ cells/L, whereas the corresponding rates in the non-cancer cohort were 1.2 and 0.9, respectively. Figure 1 shows the multivariable adjusted association of WBC count with VTE in the cancer and non-cancer cohorts. When analyzed as a continuous variable, the multivariable-adjusted HR of VTE by one standard deviation increase (SD =1.96 x 10^9^ cells/L) in WBC count was 1.19 (95% CI 1.09-1.30) in the cancer cohort and 1.00 (95% CI: 0.87-1.15) in the non-cancer cohort. In the cancer cohort, WBC count in the upper category was associated with almost 2-fold increased risk of VTE (HR 1.95, 95% CI 1.24-3.07), compared to the lowest category in the simple cox-model adjusted for age and sex. In the multivariable model, adjusted for age, sex, smoking, BMI, physical activity, self-reported diabetes mellitus and cardiovascular disease, the HR was 2.36 (95% CI 1.44-3.87)

**Table 3:** Incidence rates (IRs) and hazard ratios (HRs) for venous thromboembolism (VTE) by total white blood cell (WBC) and neutrophil counts.

	**PY** ^#^		**VTE**	IR**	**HR**†	**Multivariable HR‡**
WBC count*						
**Cancer**						
< 6.4	6613		41	6.20 (4.57-8.42)	1.00 (reference)	1.00 (reference)
6.4-8.5	6796		39	5.74 (4.19-7.86)	0.94 (0.60-1.45)	1.03 (0.66-1.62)
≥ 8.6	3400		36	10.59 (7.64-14.68)	1.95 (1.24-3.07)	2.36 (1.44-3.87)
*P for trend*					*0.01*	*0.002*
HR per 1 SD^§^	16809		116	6.90 (5.75-8.28)	1.18 (1.07-1.30)	1.19 (1.09-1.30)
**Non-cancer**						
< 6.4	97372		117	1.20 (1.00-1.44)	1.00 (reference)	1.00 (reference)
6.4-8.5	101364		110	1.09 (0.90-1.31)	0.96 (0.74-1.25)	0.91 (0.70-1.19)
≥ 8.6	49541		45	0.91 (0.68-1.22)	1.02 (0.72-1.44)	0.94 (0.65-1.36)
*P for trend*					*0.98*	*0.62*
HR per 1 SD^§^	248276		272	1.10 (0.98-1.24)	1.04 (0.91-1.18)	1.00 (0.87-1.15)
Neutrophil count*						
**Cancer**						
< 3.5		6977	44	6.31 (4.70-8.48)	1.00 (reference)	1.00 (reference)
3.5-5.0		6462	45	6.96 (5.20-9.32)	1.15 (0.76-1.74)	1.20 (0.79-1.84)
≥ 5.1		3370	27	8.01 (5.49-11.68)	1.40 (0.87-2.27)	1.56 (0.93-2.61)
*P for trend*					*0.1*	*0.09*
HR per 1 SD^§^		16809	116	6.90 (5.75-8.28)	1.26 (1.06-1.50)	1.33 (1.11-1.61)
**Non-cancer**						
< 3.5		102893	122	1.19 (1.00-1.42)	1.00 (reference)	1.00 (reference)
3.5-5.1		95624	102	1.07 (0.88-1.30)	0.94 (0.72-1.22)	0.93 (0.71-1.22)
≥ 5.1		49759	48	0.96 (0.72-1.27)	1.07 (0.76-1.49)	1.09 (0.77-1.56)
*P for trend*					*0.9*	*0.8*
HR per 1 SD^§^		248276	272	1.10 (0.98-1.24)	1.01 (0.89-1.15)	1.01 (0.88-1.16)

The Tromsø Study 1994-2007.

* 10^9^ cells/L

# Person years

** Incidence rate per 1000 person years, with 95% confidence intervals.

† Adjusted for age and sex, with 95% confidence intervals.

‡ Adjusted for age, sex, smoking, body mass index, physical activity (hard), self-reported DM and self-reported CVD, with 95% confidence intervals.

§ WBC count 1 standard deviation (SD) = 1.98, Neutrophil count 1 SD = 1.54.

Neutrophil count showed similar pattern as total WBC count in cancer and non-cancer subjects (table 3). The multivariable-adjusted HR of VTE by one standard deviation increase (SD =1.54 x 10^9^ cells/L) in neutrophil count was 1.33 (95% CI 1.11-1.61) in the cancer cohort compared to 1.01 (95% CI: 0.88-1.16) in the non-cancer cohort. In the cancer cohort, subjects with neutrophil count above the 80^th^ percentile (≥5.1 x 10^9^ cells/L) had a non-significant 1.6-fold increased risk (HR 1.56, 95% CI 0.93-2.61) of VTE compared to those with counts below the 40^th^ percentile (<3.5 x 10^9^ cells/L).

Smoking increases white blood cell count, and is also associated with cancer and VTE. Therefore, to ensure that the observed risk of VTE by WBC in cancer patients was not solely driven by a smoking induced rise in WBC count, we also conducted analysis restricted to non-smokers. In the cancer cohort, the multivariate HR for upper versus lower category of WBC count was 2.23 (95% CI 1.22-4.09), clearly indicating that the strong association was not confounded by smoking (data not shown).

The mean time from baseline to cancer diagnosis was 7.1 years. The hazard ratio of cancer did not differ significantly between the categories of WBC count when the analyses were adjusted for age, sex, BMI, smoking, physical activity, self-reported diabetes and CVD (data not shown). Thus, the observed association of leukocytes and VTE in cancer patients did not seem to be explained by an increased risk of cancer in those with a high leukocyte count.

## Discussion

This population-based cohort study is, to the best of our knowledge, the first to identify that pre-cancer leukocyte counts are associated with future risk of VTE. Cancer patients with WBC count above the 80^th^ percentile (≥ 8.6 x 10^9^ cells/L), measured on average 7 years before the cancer diagnosis, had a 2.4-fold increased risk of VTE compared to those with WBC count below the 40^th^ percentile (<6.4 x 10^9^ cells/L) in multivariable analysis. Additionally, we confirmed the original findings from the LITE study [16] showing that WBC count was not associated with risk of future VTE in the cancer-free population.

Previous studies have provided growing evidence for a predictive role of WBC count for risk of VTE in patients with overt cancer. Ambulatory cancer patients with leukocytosis (leukocyte count >11 x 10^9^ cells/L) prior to initiation of chemotherapy had a 2-fold increased risk of VTE [17]. Leukocyte count was also a risk factor for subsequent thrombosis in cancer patients receiving cisplatin [21] or gemcitabine [22] chemotherapy. Furthermore, a multicenter, VTE-registry study found that cancer patients with VTE and leukocytosis had 1.6-fold increased risk of recurrent VTE, whereas subjects with low WBC count (<4 x 10^9^ cells/L) had decreased risk of recurrence, compared to cancer patients with normal WBC count [23]. However, the design of these studies made it impossible to discriminate whether the relationship between WBC count and VTE risk in cancer patients could be causal or attributed to the presence of other cancer-related features such as co-morbidities, aggressive malignancy, the pro-inflammatory milieu or secondary to chemotherapy.

Our findings add to the present knowledge by indicating that the association between WBC count and risk of VTE observed in cancer patients is not due to WBC count as an innocent bystander of the malignant disease. First, leukocyte counts (total WBC and neutrophil counts) measured at least one year prior to cancer diagnoses, were associated with future risk of VTE in cancer patients. Second, the risk estimates for VTE by leukocyte counts measured prior to cancer development were similar to the risk estimates in a recent study where leukocyte counts were measured after cancer diagnoses [17]. Furthermore, in light of the thrombosis potential model [24], venous thrombosis will occur when a combination of risk factors reach a certain level (the thrombosis potential). Accordingly, high WBC count alone did not reach the thrombosis potential, but in combination with malignancy our findings support that WBC count contributes to reach the thrombosis threshold. Thus, the time-sequence of events and the magnitude of the risk estimates support the concept that leukocytes may be important in the pathogenesis of venous thrombosis in the presence of malignancy, but not in the general cancer-free population.

The mechanisms by which leukocytes contribute to the pathogenesis of cancer associated VTE is not clear. However, growing evidence from experimental and clinical studies indicate that microparticles expressing tissue factor (TF), shed from cancer cells and activated or apoptotic leukocytes, especially monocytes, may play an important role in the pathogenesis of cancer-related venous thrombosis [25–27]. Increased levels of P-selectin, a leukocyte adhesion molecule, expressed on activated endothelial cells and platelets [28], have been shown to increase the risk of thrombosis in cancer patients [29]. Furthermore, interaction between P-selectin and its ligand on leukocytes (P-selectin glycoprotein-1, PSGL-1) triggers the release of procoagulant microparticles [30] and promotes thrombus growth [31,32]. Recent discoveries have also proposed that neutrophils can be important in thrombus formation through formation of neutrophil extracellular traps (NETs) [33]. NETs are webs of DNA released from neutrophils during high-stress situations like sepsis, and serve as antibacterial traps [34]. NETs have been demonstrated to adhere to platelets and red blood cells, as well as to activate the coagulation cascade, thereby promoting formation of thrombin [35]. Moreover, a very recent study showed that neutrophils from mice with cancer were more prone to generate NETS than those from cancer free mice, suggesting that cancers predispose neutrophils to release extracellular DNA traps that contribute to cancer-associated thrombosis [36].

Previous population-based cohort studies have shown that leukocyte count in itself is predictive of cancer development and cancer associated mortality [15,37]. Thus, an elevated leukocyte count could represent a common underlying risk factor for cancer and VTE. In our study, however, leukocyte count was not a risk factor for cancer development. A possible explanation could be that those with a high leukocyte count at baseline developed a more aggressive type of cancer with a higher thrombotic potential. Unfortunately, due to lack of statistical power, we were not able to do sub-group analyses on risk of VTE by various cancer sites.

The main strengths of our study are the prospective design, the large population recruited from a general population, the long term follow-up, and validated exposure and endpoints. The VTE events were systematically validated and objectively confirmed, and as the University Hospital of North Norway is the only diagnostic and treating institution for VTE in the region, it is likely that the majority of the VTE events in the population were recorded. Likewise, the Cancer Registry of Norway has high completeness and validity. The Tromsø Study consists of repeated surveys (1994-95, 2001-02, 2007-08), and a subgroup of the participants was invited to participate in all the six surveys. This allows for an evaluation of variation in certain exposure variables over time. Some limitations of the study should also be mentioned. First, only one measurement of WBC count was available for the whole study population, and the risk estimates were based solely on this measurement. However in our sub-population of subjects who attended several surveys, 69% of the subjects who were initially (1994-95) categorized in the upper range of WBC count, were categorized similarly at the next measurement (2001-02). In general, intra-individual variation in an exposure variable weakens the statistical associations, and this is especially important during long-term follow-up (regression dilution effect). Furthermore, information about cancer treatment was not available in our study, and the size of the cancer cohort restricted the possibilities of sub-group analyses (e.g. cancer sites and stages).

In conclusion, leukocyte count was a risk factor for venous thrombosis in subjects who developed cancer during follow-up, but not in the cancer-free population. Our findings suggest that leukocytes may play a role in the pathogenesis of venous thrombosis in the presence of a malignant environment. 
